# The Association between Red Blood Cell Distribution Width and Sarcopenia in U.S. Adults

**DOI:** 10.1038/s41598-018-29855-z

**Published:** 2018-07-31

**Authors:** Junghoon Kim, Jeong-Soo Im, Chang Hyu Choi, Chul Hyun Park, Jae Ik Lee, Kuk Hui Son, Yoon-Hyeong Choi

**Affiliations:** 1 Department of Preventive Medicine, Gachon University College of Medicine, Incheon, Republic of Korea; 2 Department of Thoracic and Cardiovascular Surgery, Gachon University Gil Medical Centre, Gachon University College of Medicine, Incheon, Republic of Korea; 3 Gachon Advanced Institute for Health Sciences and Technology, Gachon University, Incheon, Republic of Korea

## Abstract

One pathophysiological sign of sarcopenia is chronic inflammation. Given that levels of red blood cell distribution width (RDW) are increased in chronic inflammation, we evaluated the association between increased RDW and sarcopenia among adults in the general U. S. population and analyzed data from 11,761 participants from the National Health and Nutrition Examination Survey (NHANES) 1999–2006. Sarcopenia was defined as an appendicular skeletal muscle mass (ASM) divided by weight (%) that was less than one standard deviation (SD) below the mean of young adults. The odds ratios (ORs) and confidence intervals (CIs) for sarcopenia were calculated across RDW quartiles after adjusting for confounding factors. Elevated RDW levels were significantly associated with sarcopenia after adjusting for age, sex, race, education, household income, smoking, physical activity, hypertension, diabetes, cardiovascular disease, C-reactive protein, and hemoglobin (OR of highest quartile: 1.72 (95% CI: 1.43, 2.06)). Further, in a model stratified by obesity, an elevated RDW was associated with sarcopenia in the overweight and obese group, but not in the normal weight group. Our study shows that elevated RDW is associated with sarcopenia, and this association is particularly strong in people who are overweight and obese.

## Introduction

Sarcopenia is characterized by the loss of muscle mass and the decline of muscle function that occurs with aging^1^. Sarcopenia is known to be related to having fallen and decreased physical ability and is highly prevalent in the elderly^1–6^. Sarcopenia has become a major health problem at the individual as well as community levels, and it is a challenge to prevent or delay sarcopenia.

Although the underlying mechanisms in sarcopenia are not fully understood, inflammation, neuromuscular, and hormonal changes as well as nutrition and physical inactivity are currently discussed as leading to sarcopenia^1,7,8^.

Red blood cell distribution width (RDW) is a simple laboratory parameter that indicates the variability in the size of circulating erythrocytes^9^. Increased RDW values have been reported in relationship to underlying chronic inflammation which induces red blood cell (RBC) membrane deformability and changes in erythropoiesis^10^. In fact, an epidemiologic study has suggested that RDW is associated with increased levels in high-sensitivity C-reactive protein (CRP), a biomarker of inflammation^10^. Further, recent epidemiologic studies have reported that RDW may be an effective predictor of chronic diseases and mortality in cardiovascular disease (CVD), cancer, and other diseases^11–16^. Accordingly, we hypothesized that chronic inflammation may be the common pathophysiological link between increased RDW levels and sarcopenia. Thus, we evaluated the association between increased RDW and the risk of sarcopenia in a nationally representative U.S. population using the National Health and Nutrition Examination Survey (NHANES) 1999–2006.

## Results

Table 1 shows participant characteristics in subjects with non-sarcopenia and sarcopenia. In the overall participants, mean age (±standard error, SE) was 45.30 (±0.25) years and mean skeletal muscle index (ASM) (±SE) was 27.65 (±0.07) % with a mean RDW (±SE) of 12.59 (±0.01) %. Participants with sarcopenia tend to be older, male, to have higher body mass index (BMI), CRP, hemoglobin and RDW, and lower moderate-to-vigorous physical activity (MVPA). Moreover, the prevalence of hypertension, diabetes, and CVD was significantly higher in participants with sarcopenia compared to non-sarcopenic participants.

**Table 1:** Participant characteristics in subjects with non-sarcopenia and sarcopenia.

	Overall (n = 11761)		Non-sarcopenia (n = 7880)		Sarcopenia (n = 3881)		*P-value*
Age [years]^*a*^	45.30	±0.25	42.06	±0.26	53.19	±0.33	<*0*.*001*
BMI [kg/m^2^]	27.85	±0.09	26.18	±0.08	31.90	±0.13	<*0*.*001*
CRP [mg/dL]	0.40	±0.01	0.31	±0.01	0.60	±0.02	<*0*.*001*
Hemoglobin [g/dL]	14.56	±0.04	14.53	±0.04	14.64	±0.05	<*0*.*001*
ASM [%]	27.65	±0.07	29.03	±0.07	24.29	±0.07	<*0*.*001*
RDW [%]	12.59	±0.01	12.51	±0.02	12.79	±0.02	<*0*.*001*
Sex [n (%)]^*b*^							
Male	5965	(49.5)	3896	(48.4)	2069	(52.3)	*0*.*002*
Female	5796	(50.5)	3984	(51.6)	1812	(47.7)	
Race/ethnicity [n (%)]							
Non-Hispanic White	6123	(73.9)	3868	(71.6)	2255	(79.5)	<*0*.*001*
Non-Hispanic Black	2241	(9.8)	1949	(12.3)	292	(3.7)	
Mexican American	2509	(6.7)	1420	(6.1)	1089	(8.0)	
Other	888	(9.6)	643	(10.0)	245	(8.8)	
Education [n (%)]							
<High School	3382	(17.5)	2051	(16.2)	1331	(20.8)	<*0*.*001*
High School	2804	(25.6)	1828	(24.3)	976	(29.0)	
>High School	5575	(56.8)	4001	(59.6)	1574	(50.2)	
Household income [n (%)]							
PIR <1	1938	(11.8)	1306	(12.0)	632	(11.4)	*0*.*017*
PIR 1 to <3	4843	(35.2)	3081	(34.0)	1762	(38.2)	
PIR ≥3	4980	(52.9)	3493	(54.0)	1487	(50.4)	
Smoking pack-years							
Never	6484	(54.3)	4513	(56.3)	1971	(49.4)	<*0*.*001*
<20	3559	(31.7)	2515	(33.3)	1044	(28.0)	
≥20	1718	(14.0)	852	(10.5)	866	(22.6)	
MVPA [n (%)]							
Low	7751	(61.2)	4853	(56.6)	2898	(72.1)	<*0*.*001*
High	4010	(38.8)	3027	(43.4)	983	(27.9)	
Diabetes [n (%)]							
No	10473	(92.6)	7314	(95.5)	3159	(85.6)	<*0*.*001*
Yes	1288	(7.4)	566	(4.5)	722	(14.4)	
Hypertension [n (%)]							
No	7442	(70.4)	5620	(78.1)	1822	(51.9)	<*0*.*001*
Yes	4319	(29.6)	2260	(21.9)	2059	(48.1)	
CVD [n (%)]							
No	10547	(92.4)	7342	(95.2)	3205	(85.6)	<*0*.*001*
Yes	1214	(7.6)	538	(4.8)	676	(14.4)	

^*a*^Weighted mean ± SE from survey mean (all such values).

^*b*^Weighted percentages from survey frequency (all such values).

*P*-values were calculated using *t*-tests for continuous variables and Rao-Scott chi-square tests for categorical variable according to survey procedures.

BMI, body mass index; CRP, C-reactive protein; ASM, appendicular skeletal muscle mass; RDW, red blood cell distribution width; PIR, poverty income ratio; MVPA, moderate-to-vigorous physical activity; CVD, cardiovascular disease.

Table 2 shows characteristics of the study population by quartiles of RDW. Participants with a higher RDW tended to be older and female, and to have a higher BMI and CRP levels, lower ASM and hemoglobin, higher amount of smoking pack-years, less MVPA, and a higher prevalence of hypertension, diabetes, and CVD.

**Table 2:** Characteristics of study population by red blood cell distribution width.

Characteristics	Red blood cell distribution width								*P-value*
	Q1 (10.5–12.0%) (n = 2676)		Q2 (12.1–12.4%) (n = 2732)		Q3 (12.5–13.0%) (n = 3315)		Q4 (13.1–31.6%) (n = 3038)		
Age [years]^*a*^	40.70	±0.36	44.25	±0.38	47.16	±0.34	50.44	±0.38	<*0*.*001*
BMI [kg/m^2^]	26.58	±0.13	27.38	±0.14	28.45	±0.15	29.36	±0.17	<*0*.*001*
CRP [mg/dL]	0.29	±0.01	0.35	±0.02	0.40	±0.01	0.60	±0.02	<*0*.*001*
Hemoglobin [g/dL]	14.76	±0.05	14.84	±0.05	14.64	±0.04	13.81	±0.05	<*0*.*001*
ASM [%]	28.22	±0.13	28.23	±0.10	27.41	±0.12	26.45	±0.12	<*0*.*001*
RDW [%]	11.74	±0.01	12.26	±0.003	12.69	±0.003	14.06	±0.03	<*0*.*001*
Sex [n (%)]^*b*^									
Male	1335	(48.1)	1519	(55.2)	1742	(51.2)	1369	(42.1)	<*0*.*001*
Female	1341	(51.9)	1213	(44.8)	1573	(48.8)	1669	(57.9)	
Race/ethnicity [n (%)]									
Non-Hispanic White	1595	(79.0)	1560	(77.2)	1707	(73.5)	1261	(63.4)	<*0*.*001*
Non-Hispanic Black	270	(4.9)	382	(6.8)	608	(10.1)	981	(19.8)	
Mexican American	590	(6.6)	583	(6.5)	757	(7.1)	579	(6.3)	
Other	221	(9.4)	207	(9.5)	243	(9.3)	217	(10.5)	
Education [n (%)]									
<High School	603	(13.6)	702	(15.5)	1014	(18.7)	1063	(23.9)	<*0*.*001*
High School	608	(23.2)	649	(25.4)	823	(28.0)	724	(26.1)	
>High School	1465	(63.1)	1381	(59.0)	1478	(53.3)	1251	(50.1)	
Household income [n (%)]									
PIR <1	368	(9.6)	406	(10.9)	570	(12.0)	594	(16.0)	<*0*.*001*
PIR 1 to <3	1021	(32.5)	1063	(33.3)	1379	(35.9)	1380	(40.5)	
PIR ≥3	1287	(58.0)	1263	(55.8)	1366	(52.1)	1064	(43.6)	
Smoking pack-years									
Never	1577	(58.3)	1543	(54.6)	1777	(52.5)	1587	(50.6)	<*0*.*001*
<20	829	(31.9)	840	(32.9)	1026	(32.5)	864	(29.1)	
≥20	270	(9.8)	349	(12.5)	512	(15.0)	587	(20.3)	
MVPA [n (%)]									
Low	1597	(55.1)	1713	(59.2)	2207	(62.6)	2234	(70.0)	<*0*.*001*
High	1079	(44.9)	1019	(40.8)	1108	(37.4)	804	(30.0)	
Diabetes [n (%)]									
No	2490	(95.2)	2473	(93.7)	2955	(92.6)	2555	(87.9)	<*0*.*001*
Yes	186	(4.8)	259	(6.3)	360	(7.4)	483	(12.1)	
Hypertension [n (%)]									
No	1985	(78.1)	1904	(74.7)	1994	(66.6)	1559	(59.7)	<*0*.*001*
Yes	691	(21.9)	828	(25.3)	1321	(33.4)	1479	(40.3)	
CVD [n (%)]									
No	2543	(96.5)	2512	(93.5)	2962	(91.8)	2530	(86.3)	<*0*.*001*
Yes	133	(3.5)	220	(6.5)	353	(8.2)	508	(13.7)	

^*a*^Weighted mean ± SE from survey mean (all such values).

^*b*^Weighted percentages from survey frequency (all such values).

*P* for trend was calculated using linear regression for continuous variables; *P* for difference was calculated using Rao-Scott chi-square test for categorical variables.

BMI, body mass index; CRP, C-reactive protein; ASM, appendicular skeletal muscle mass; RDW, red blood cell distribution width; PIR, poverty income ratio; MVPA, moderate-to-vigorous physical activity; CVD, cardiovascular disease.

Table 3 shows odds ratios (ORs) and 95% confidence intervals (CIs) by quartiles of RDW. When we examined sarcopenia that was defined using lower muscle mass alone, RDW levels were significantly associated with the risk of sarcopenia in all sequential models (all *P* for trend <0.001). In model A, the ORs for sarcopenia were 1.48 (95% CI: 1.28, 1.70) in participants in the third quartile and 1.93 (95% CI: 1.60, 2.34) in participants in the highest quartile compared with the lowest quartile of RDW, after adjusted for age, sex, race/ethnicity, education, and household income. These results did not change after additionally adjusting for smoking pack-years and MVPA in Model B, and adjusting for hypertension, diabetes, and CVD in model C. In fully adjusted model (Model D), the ORs for sarcopenia were 1.41 (95% CI: 1.23, 1.61) in participants in the third quartile and 1.72 (95% CI: 1.43, 2.06) in participants in the highest quartile compared with the lowest quartile of RDW.

**Table 3:** OR (95% CIs) for sarcopenia by red blood cell distribution width.

Red blood cell distribution width	Sarcopenia/Participants	Model A		Model B		Model C		Model D	
		OR	95% CI	OR	95% CI	OR	95% CI	OR	95% CI
**Sarcopenia defined using appendicular skeletal muscle mass** ^***a***^									
Q1 (10.5–12.0%)	641/2676	1.00	(Reference)	1.00	(Reference)	1.00	(Reference)	1.00	(Reference)
Q2 (12.1–12.4%)	786/2732	1.01	(0.90, 1.14)	1.00	(0.89, 1.13)	1.01	(0.90, 1.13)	0.98	(0.87, 1.10)
Q3 (12.5–13.0%)	1220/3315	1.48	(1.28, 1.70)	1.45	(1.27, 1.67)	1.45	(1.27, 1.66)	1.41	(1.23, 1.61)
Q4 (13.1–31.6%)	1234/3038	1.93	(1.60, 2.34)	1.85	(1.54, 2.22)	1.80	(1.50, 2.17)	1.72	(1.43, 2.06)
*P for trend*		<*0*.*001*		<*0*.*001*		<*0*.*001*		<*0*.*001*	
**Sarcopenia defined using both appendicular skeletal muscle mass** ^***a***^ **and walking speed** ^***b***^									
Q1 (10.5–12.0%)	37/481	1.00	(Reference)	1.00	(Reference)	1.00	(Reference)	1.00	(Reference)
Q2 (12.1–12.4%)	55/592	0.84	(0.51, 1.39)	0.83	(0.50, 1.37)	0.77	(0.48, 1.25)	0.77	(0.46, 1.28)
Q3 (12.5–13.0%)	106/888	0.98	(0.63, 1.53)	0.96	(0.62, 1.49)	0.94	(0.60, 1.48)	0.92	(0.58, 1.45)
Q4 (13.1–31.6%)	160/864	1.73	(1.08, 2.75)	1.66	(1.04, 2.65)	1.67	(1.06, 2.63)	1.58	(1.00, 2.52)
*P for trend*		*0*.*019*		*0*.*024*		*0*.*022*		*0*.*038*	

^*a*^Low muscle mass defined as appendicular skeletal muscle mass <29.36% for males and <22.41% for females.

^*b*^Decline physical function defined as walking speed ≤0.8 m/s.

^*b*^Data for these analyses (n = 2,825) used subpopulation (≥50 years) that had walking speed information.

Model A: adjusted for age, sex, race/ethnicity, education, and household income.

Model B: adjusted for Model A covariates plus smoking pack-years and moderate-to-vigorous physical activity.

Model C: adjusted for Model B covariates plus hypertension, diabetes, and cardiovascular disease.

Model D: adjusted for Model C covariates plus C-reactive protein and hemoglobin.

When we examined sarcopenia that was defined using both lower muscle mass and lower walking speed, we also found that higher RDW levels were significantly associated with an increased OR for sarcopenia after fully adjusting for covariates (OR: 1.58, 95% CI: 1.00, 2.52 for the highest vs. lowest quartile of RDW, *P* for trend = 0.038). These results were similar to those using the definition of sarcopenia with lower muscle mass alone.

We examined variance inflation factors (VIFs) to detect any multicollinearity between the predictors in model D, but found no statistical significance (all VIFs were <10).

We conducted sensitivity analyses after additional adjustment for BMI (Table S1). We found that the associations of RDW with sarcopenia remained after additionally adjusting for BMI, although the effects of RDW on the risk of sarcopenia was weakened.

**Figure 1:**
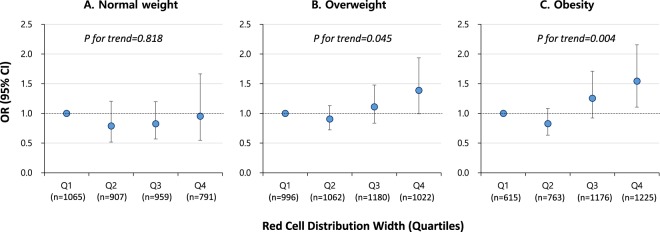
OR (95% CIs) for sarcopenia by red blood cell distribution width by obesity status. Adjusted for age, sex, race/ethnicity, education, household income, smoking pack-years, moderate-to-vigorous physical activity, hypertension, diabetes, cardiovascular disease, C-reactive protein, and hemoglobin (Model D in Table 3). *P* for interaction by obesity status = 0.748.

We examined stratified models by obesity status (normal weight, overweight, and obesity; Fig. 1). There was no significant association between RDW and the risk of sarcopenia among participants with normal weight (*P* for trend = 0.818, Fig. 1A). In contrast, participants with higher RDW showed a significantly increased OR for sarcopenia in the overweight group (*P* for trend = 0.045, Fig. 1B) and obese group (*P* for trend = 0.004, Fig. 1C). Yet we observed no statistically significant effect modification by obesity status (*P* for interaction = 0.748). When we examined participant characteristics by obesity status, those subjects at greater weight had significantly higher age, BMI, CRP, and RDW, and lower ASM. Further, other variables (sex, race/ethnicity, education, income, smoking, MVPA, and presence of diabetes, hypertension, and CVD) except hemoglobin were significantly different depending on obesity status (Table S2).

We conducted another sensitivity analysis for the association of RDW with sarcopenic obesity and found a strong association (Table S3). This was consistent with the associations of sarcopenia shown in Table 3.

Moreover, we examined stratified models by age groups (20–39, 40–59, and ≥60 years). There were no significant associations between RDW and sarcopenia or sarcopenic obesity across the age strata (Table S4).

## Discussion

Sarcopenia is a systemic condition with various risk factors and whose prevalence increases with age^17^. Previous studies have shown that sex is an inconclusive risk factor (e.g., Landi *et al*. found that sarcopenia was more risky in men^18^, whereas Yu *et al*. reported that it was more risky in women^19^), and that sarcopenia is related to lifestyle factors such as smoking, obesity, and low physical activity^20^. Sarcopenia is associated with elevated levels of markers of inflammation such as CRP and interleukin (IL)-6^21^, and the prevalence of sarcopenia is higher in patients with other disease such as chronic obstructive pulmonary disorder, hypertension, diabetes, and CVD^22^. Results from the present study support the previous studies: compared to subjects who were not sarcopenic, subjects with sarcopenia were older, were likely to be heavy smokers, and to have hypertension, diabetes, CVD, and low physical activity.

The aim of the present study was to investigate the associations between elevated RDW and sarcopenia, after controlling for potential confounders. We found that RDW levels were significantly associated with the risk of sarcopenia.

Increased RDW reflects greater heterogeneity in RBC volume^9^. Disorders related to ineffective RBC production (e.g., iron deficiency anemia, vitamin B12 and folic acid deficiency, bone marrow suppression, and hemoglobinopathies), to increased RBC destruction (hemolysis), or to blood transfusion can all induce elevation in RDW^9,11^. Elevated RDW is related to chronic disease as well as to RBC production function or bone marrow function.

Independent of hemoglobin level, RDW is associated with diabetes^14^ and is one of the strongest independent predictors of morbidity and mortality in various cardiovascular conditions that include coronary heart disease, pulmonary hypertension, acute heart failure, peripheral artery disease, stroke, or pulmonary embolism^9,12,13,16,23–25^.

Although the definitive mechanism underlying relationships between RDW and these diseases remains unclear, several potential mechanisms may be suggested from previous reports. First, bone marrow function and iron metabolism may be influenced by the systemic inflammation response^9,26,27^. Second, erythropoietin-induced erythrocyte maturation and proliferation are restrained by pro-inflammatory cytokines^9,28^. Third, pro-inflammatory cytokines usually down-regulate the expression of erythropoietin receptor, important in the process of erythrocyte maturation^9,28^, resulting in the release of larger, immature reticulocytes into circulation^9^. Lastly, RDW is significantly influenced by biomarkers of inflammation such as CRP, erythrocyte sedimentation rate (ESR), white blood count (WBC), and IL-6^9,29–32^.

Many previous studies have shown that inflammatory cytokines activate various molecular pathways involved in muscle wasting and lead to an imbalance between protein synthesis and catabolism^33–35^. Sarcopenia is related to elevation in levels of inflammation markers, such as CRP, IL-6^21^, and tumor necrosis factor (TNF)-α^36^. Moreover, a large cohort study suggested that RDW is a strong marker of inflammatory activity, such as hs-CRP and ESR^10^. Therefore, the association between RDW and sarcopenia in the present study might be due to shared pathophysiology (i.e., inflammation).

Further, we examined models stratified by status of obesity. Interestingly, there was no association between RDW and sarcopenia in the normal weight group, while elevated RDW was associated with sarcopenia in the overweight and obesity groups, although the effect modification by obesity status was not significant. In sensitivity analysis, we compared participant characteristics in those three groups (normal weight, overweight, and obesity) and observed higher RDW levels but lower ASM among the overweight and obesity groups. Obesity is currently recognised as a subclinical inflammatory state^32^. Our study shows that obesity plays a role in triggering the association between elevated RDW and sarcopenia. These results suggest that chronic inflammation may be a link between RDW and sarcopenia, aggravated by obesity.

Although sarcopenia can be defined generally in the loss of muscle mass, research groups such as European Working Group on Sarcopenia (EWGS) have recently suggested that a reduction in muscle function needs to be considered in defining sarcopenia — that is, that sarcopenia should be defined using both low muscle mass (a measure of ASM using dual-energy X-ray absorptiometry (DXA)) and low muscle function (a physical measure of walking speed and/or handgrip strength)^1,37^. Therefore, in the present study, we used two different definitions of sarcopenia as follows: a commonly used definition (i.e., a crude definitions) and a recently suggested definition (i.e., a robust definition). We showed that the association of RDW with sarcopenia was consistent between the two different definitions.

There are several limitations in the present study. First, we cannot identify a primary sarcopenia (i.e., age-related sarcopenia) and secondary sarcopenia (i.e., related to other medical conditions) due to non-available information in NHANES, although the pathophysiological nature of primary and secondary sarcopenia are different. Second, NHANES is a cross-sectional study, thus we cannot infer a causal relationship between RDW and sarcopenia. Therefore, we cannot conclude that RDW is a risk factor for development of sarcopenia, and there is a possibility that elevated RDW is a consequence of sarcopenia. Despite these limitations, to our knowledge, this is the first investigation of the associations of RDW and the risk of sarcopenia. The strengths of this study include the use of a representative sample of the general adult population and objective measures of skeletal muscle mass and function, as well as adjustment for various potential confounders. RDW can be measured using a simple and inexpensive blood test. If further study confirms that RDW has a causal relationship in sarcopenia, RDW can be used as a marker to predict sarcopenia.

In conclusion, the current study shows that elevated RDW is associated with the risk of sarcopenia in a representative sample of the U.S. general adult population. That association was particularly strong in overweight and obese participants, whereas no association was found in participants with normal weight.

## Methods

### Study population

NHANES is an ongoing cross-sectional survey of a nationally representative U.S. population conducted by the CDC’s National Center for Health Statistics. This survey includes an initial extensive interview at home with a subsequent physical examination and additional interviews at a mobile examination center (MEC)^38^.

The present study used data from NHANES 1999–2006 on participants who were 20 years or older and who had DXA measures (no DXA measurement was made of NHANES subjects who were pregnant, had height >192.5 cm (>6′5″), or had weight >136.4 kg (>300 lbs)). There were 14,943 participants with DXA measures in the initial eligible sample. For this analysis, we excluded participants with invalid data for muscle mass even if they had completed the DXA measures (n = 10). Of those 14,933 adults with valid DXA measures, only 14,467 had data on blood counts. After further excluding participants with data missing for clinical health condition (n = 502), CRP (n = 134), BMI (n = 131), lifestyle factors (n = 1,040), and socio demographic factors (n = 899), 11,761 adults were included in the sample for analysis. The NHANES study was approved by the Institutional Review Board (IRB) of the National Center for Health Statistics (NCHS), and all participants provided written informed consent. All methods were carried out in accordance with relevant guidelines and regulations.

### Sarcopenia and sarcopenic obesity

When we diagnosed sarcopenia, we used two different definitions as follows: (1) using ASM (i.e., lower muscle mass) and (2) using ASM and physical function (i.e., lower muscle mass and lower walking speed). When we used the latter definition, the sample size for the analyses was small (n = 2,825) because data on walking speed were available only in a subset of NHANES.

Skeletal muscle mass was determined for each participant using DXA scans (QDR-4500 Hologic scanner, Bedford, MA)^39^. Muscle mass was calculated as the sum of the appendicular lean mass, and ASM was computed in dividing appendicular lean mass by body weight (appendicular skeletal muscle (kg)/body weight (kg) × 100). In order to calculate cut-off values of ASM, we selected a reference group of young participants aged 20–39 years who had completed DXA measures (2,043 males and 1,891 females). Lower muscle mass was defined when the ASM values were less than the value of sex-specific mean minus one standard deviation (SD) of young reference group^5^. The mean ± SD of ASM in the reference group was 32.66 ± 3.30 for male and 25.44 ± 3.02 for female. In this study, the cut-off values of ASM to define lower muscle mass was 29.36% (kg/kg × 100) for males and 22.41% (kg/kg × 100) for females, respectively.

Physical function was estimated from walking speed that was available in a subsample of participants (n = 2,825). Time to complete a 20-ft walk test was measured at their usual pace in participants ≥50 years of age. We computed walking speed (m/s) by dividing the walked distance (6.096 m) by measured time to walk (seconds), and then defined lower physical function as walking speed of ≤0.8 m/s, as recommended by EWGS^1^.

Sarcopenic obesity was referred to a combination of sarcopenia and obesity. Sarcopenia was defined using two different definitions as described above, and obesity was defined as BMI ≥ 30 kg/m^2^. BMI (kg/m^2^) was computed as body weight (kg) divided by height squared (m^2^).

### Red Blood Cell Distribution Width

Whole blood samples were collected from all participants, and RBCs were measured using the Beckman automated Coulter method of counting and sizing (Coulter Counter; Coulter Electronics, Luton, UK)^40^. Mean Corpuscular Volume (MCV) was calculated as the average volume of individual erythrocytes derived from the histogram of RBC. Finally, the RDW (%) was calculated as the SD of MCV divided by the mean of MCV, multiplied by 100. Because the distribution of RDW was highly skewed, we did not model RDW as a continuous variable, but as a categorical variable instead.

### Other covariates

Confounding factors included age, sex, race/ethnicity, education level, household income, smoking pack-years, MVPA, hypertension, diabetes, CVD, CRP, and hemoglobin.

Race/ethnicity was categorized into four groups as Non-Hispanic White, Non-Hispanic Black, Mexican American, and Other. Education level was categorized as <high school, high school, and >high school. Household income was calculated by dividing family income by a poverty income ratio (PIR) threshold that was specific to family size and then categorized into three groups as <1 (below poverty line), 1 to <3, and ≥3. Cumulative smoking pack-year was categorized as never, <20 and ≥20 pack-years. MVPA was self-reported for moderate-to-vigorous activities performed in leisure-time over the previous 30 days. Based on recommendation for physical activity from the 2008 Physical Activity Guidelines for Americans^41^, participants who reported participating at least 150 minutes a week of moderate-intensity, 75 minutes a week of vigorous-intensity physical activity, or an equivalent combination of moderate- and vigorous-intensity activity, were defined as high MVPA.

Hypertension was defined as systolic blood pressure reading ≥140 mmHg or diastolic blood pressure ≥90 mmHg, current use of anti-hypertensive medication, or self-reported physician diagnosis. Type 2 diabetes mellitus was defined as fasting plasma glucose level ≥126 mg/dL, current use of anti-hyperglycemia medication, or self-reported physician diagnosis. CVD was defined as a self-reported presence of coronary heart disease, heart attack, congestive heart failure, angina/angina pectoris, or stroke.

In sensitivity analyses, BMI was considered as an additional potential confounder.

### Statistical analysis

To account for the complex survey design, all statistical analyses were performed using SAS survey procedures (ver. 9.4, SAS Institute, Cary, NC). Participant characteristics were described as weighted means and SE for continuous variables and as weighted percentage for categorical variables. We used PROC SURVEYLOGISTIC to estimate the OR and 95% CI for sarcopenia by RDW concentrations as quartiles. We also computed *P*-value for linear trend by fitting the quartile of RDW as an ordinal categorical variable, coded using integer values (0–3). To examine the influence of potential confounders, we developed four sequential models: model A was adjusted for age, sex, race/ethnicity, education, and household income; model B was additionally adjusted for smoking pack-years and MVPA; model C was further adjusted for hypertension, diabetes, and CVD; model D was further adjusted for CRP and hemoglobin concentrations. We also tested the VIFs for multicollinearity between the predictors in the fully adjusted model^42^.

In order to evaluate obesity-related differences in the associations between RDW and sarcopenia, we additionally examined stratified models by obesity status (normal weight (BMI < 25 kg/m^2^), overweight (25 ≤ BMI < 30 kg/m^2^), and obesity (BMI ≥ 30 kg/m^2^).

### Sensitivity analysis

First, we conducted sensitivity analyses after further adjusting for BMI as a potential confounder, because obesity is linked to lower muscle mass^43^ and associated with elevated RDW levels^44^. Second, we conducted sensitivity analysis to estimate the ORs and 95% CIs for sarcopenic obesity by RDW levels. Finally, we performed sensitivity analysis of the associations between RDW and sarcopenia or sarcopenic obesity using stratified models by age groups (20–39, 40–59, and ≥60 years).

## Electronic supplementary material

Table S1, Table S2, Table S3, Table S4
