# Supplementary material for: The Association between Red Blood Cell Distribution Width and Sarcopenia in U.S. Adults
Source: Sci Rep. 2018 Jul 31;8:11484. doi: 10.1038/s41598-018-29855-z (PMC6068096; doi:10.1038/s41598-018-29855-z)
Supplement: Supplementary file 1 — Table S1, Table S2, Table S3, Table S4 [file 41598_2018_29855_MOESM1_ESM.pdf]

The Association between Red Blood Cell Distribution Width and Sarcopenia in U.S. Adults

Junghoon Kim[1], Jeong-Soo Im[1], Chang Hyu Choi[2], Chul Hyun Park[2], Jae Ik Lee[2], Kuk Hui Son[2]*, Yoon-Hyeong Choi[1,3]*

Authors Affiliation

[1] *Department of Preventive Medicine, Gachon University College of Medicine, Incheon, Republic of Korea*

[2] *Department of Thoracic and Cardiovascular Surgery, Gachon University Gil Medical Centre, Incheon, Republic of Korea*

[3] *Gachon Advanced Institute for Health Sciences and Technology, Gachon University, Incheon, Republic of Korea*

*** Corresponding author:**

Kuk Hui Son, MD, PhD,
Department of Thoracic and Cardiovascular Surgery,
Gachon University Gil Medical Centre,
21 Namdong-daero 774beon-gil, Namdong-gu,
Incheon, Republic of Korea 21565
Phone: +82-32-460-2783
Fax: +82-32-899-6039
E-mail: dr632@gilhospital.com

*** Corresponding author:**

Yoon-Hyeong Choi, PhD,
Department of Preventive Medicine,
Gachon University College of Medicine,
155 Gaetbeol-ro, Yeonsu-gu,
Incheon, Republic of Korea 21999
Phone: +82-32-899-6586
Fax: +82-32-468-2154
E-mail: yoonchoi@gachon.ac.kr

Supplementary Material

Supplemental Material, Table S1. Body mass index-adjusted OR (95% CIs) for sarcopenia by red cell distribution width

Red cell distribution width	Sarcopenia/ Participants	Non-adjustment[a] for body mass index		Adjustment[b] for body mass index	
		OR	(95% CI)	OR	(95% CI)
Q1 (10.5-12.0 %)	641/2676	1.00	(Reference)	1.00	(Reference)
Q2 (12.1-12.4 %)	786/2732	0.98	(0.87, 1.10)	0.83	(0.73, 0.94)
Q3 (12.5-13.0 %)	1220/3315	1.41	(1.23, 1.61)	1.04	(0.89, 1.22)
Q4 (13.1-31.6 %)	1234/3038	1.72	(1.43, 2.06)	1.23	(0.97, 1.55)
P for trend		*<0.001*		*0.046*	

[a]Adjusted for age, sex, race/ethnicity, education, household income, smoking pack-years, moderate-to-vigorous physical activity, hypertension, diabetes, cardiovascular disease, C-reactive protein, and hemoglobin (Model D in Table 3).
[b]Further adjusted for body mass index.

Supplemental Material, Table S2. Characteristics of study population by obesity status

Characteristics	Normal weight (n=3722)		Overweight (n=4260)		Obesity (n=3779)		*P-value*
Age [years] [a]	42.32	±0.32	46.90	±0.33	46.84	±0.35	*<.001*
BMI [kg/m^2]	22.19	±0.04	27.41	±0.03	34.67	±0.08	*<.001*
CRP [mg/dL]	0.24	±0.01	0.37	±0.01	0.59	±0.02	*<.001*
Hemoglobin [g/dL]	14.4	±0.05	14.8	±0.04	14.5	±0.04	*0.158*
ASM [%]	29.34	±0.12	28.07	±0.10	25.29	±0.08	*<.001*
RDW [%]	12.47	±0.02	12.55	±0.02	12.79	±0.03	*<.001*
Sex [n (%)][b]							
Male	1782	(43.0)	2527	(59.6)	1656	(45.5)	*<.001*
Female	1940	(57.0)	1733	(40.4)	2123	(54.5)	
Race/ethnicity [n (%)]							
Non-Hispanic White	2142	(76.2)	2171	(73.3)	1810	(72.1)	*<.001*
Non-Hispanic Black	605	(7.9)	732	(8.9)	904	(12.9)	
Mexican American	642	(5.4)	1033	(7.6)	834	(7.0)	
Other	333	(10.5)	324	(10.2)	231	(8.0)	
Education [n (%)]							
<High School	972	(16.2)	1286	(18.1)	1124	(18.4)	*<.001*
High School	851	(23.4)	992	(25.7)	961	(28.1)	
>High School	1899	(60.4)	1982	(56.2)	1694	(53.5)	
Household income [n (%)]							
PIR <1	646	(13.1)	665	(10.2)	627	(12.3)	*<.001*
PIR 1 to <3	1460	(33.7)	1744	(34.6)	1639	(37.6)	
PIR ≥3	1616	(53.2)	1851	(55.2)	1513	(50.2)	
Smoking pack-years							
Never	2009	(54.2)	2312	(53.1)	2163	(55.6)	*<.001*
<20	1210	(34.2)	1297	(31.9)	1052	(28.8)	
≥20	503	(11.5)	651	(15.0)	564	(15.6)	
MVPA [n (%)]							
Low	2311	(56.7)	2766	(59.6)	2674	(68.0)	*<.001*
High	1411	(43.3)	1494	(40.4)	1105	(32.0)	
Diabetes [n (%)]							
No	3516	(96.8)	3800	(93.3)	3157	(87.2)	*<.001*
Yes	206	(3.2)	460	(6.7)	622	(12.8)	
Hypertension [n (%)]							
No	2811	(83.0)	2640	(69.3)	1991	(57.7)	*<.001*
Yes	911	(17.0)	1620	(30.7)	1788	(42.3)	
CVD [n (%)]							
No	3434	(95.2)	3817	(92.2)	3296	(89.5)	*<.001*
Yes	288	(4.8)	443	(7.8)	483	(10.5)	

[a] Weighted mean±SE from survey mean (all such values).
[b] Weighted percentages from survey frequency (all such values).
P for trend was calculated using linear regression for continuous variables; *P* for difference was calculated using Rao-Scott chi-square test for categorical variables.
BMI, body mass index; CRP, C-reactive protein; ASM, appendicular skeletal muscle mass; RDW, red cell distribution width; PIR, poverty income ratio; MVPA, moderate-to-vigorous physical activity; CVD, cardiovascular disease.

Supplemental Material, Table S3. OR (95% CIs) for sarcopenic obesity by red cell distribution width

Red blood cell distribution width	Sarcopenic obesity/Participants	OR	95% CI
Sarcopenic obesity defined using appendicular skeletal muscle mass[a] plus body mass index[b]			
Q1 (10.5-12.0 %)	321/2676	1.00	(Reference)
Q2 (12.1-12.4 %)	395/2732	1.15	(0.92, 1.42)
Q3 (12.5-13.0 %)	685/3315	1.83	(1.49, 2.23)
Q4 (13.1-31.6 %)	710/3038	2.22	(1.79, 2.77)
P for trend			*<0.001*
Sarcopenic obesity defined using both appendicular skeletal muscle mass[a] and walking speed[c] plus body mass index[b]			
Q1 (10.5-12.0 %)	14/481	1.00	(Reference)
Q2 (12.1-12.4 %)	29/592	1.89	(0.66, 5.42)
Q3 (12.5-13.0 %)	42/888	1.27	(0.46, 3.54)
Q4 (13.1-31.6 %)	83/864	3.50	(1.26, 9.70)
P for trend			*0.019*

[a]Low muscle mass defined as appendicular skeletal muscle mass <29.36% for males and <22.41% for females.
[b]Obesity defined as body mass index ≥30kg/m^2.
[c]Decline physical function defined as walking speed ≤0.8 m/s.
[c]Data for these analyses (n=2,825) used subpopulation (≥50 years) that had walking speed information.
Adjusted for age, sex, race/ethnicity, education, household income, smoking pack-years, moderate-to-vigorous physical activity, hypertension, diabetes, cardiovascular disease, C-reactive protein, and hemoglobin (Model D in Table 3).

Supplemental Material, Table S4. OR (95% CIs) for sarcopenia and sarcopenic obesity by red cell distribution width by age group

Red blood cell distribution width	Sarcopenia or sarcopenic obesity/ Participants	Overall		Age: 20-39 years		Age: 40-59 years		Age: ≥60 years	
		OR	95% CI	OR	95% CI	OR	95% CI	OR	95% CI
Sarcopenia defined using appendicular skeletal muscle mass[a]									
Q1 (10.5-12.0 %)	641/2676	1.00	(Reference)	1.00	(Reference)	1.00	(Reference)	1.00	(Reference)
Q2 (12.1-12.4 %)	786/2732	0.98	(0.87, 1.10)	0.99	(0.75, 1.31)	0.82	(0.68, 0.99)	1.24	(0.97, 1.58)
Q3 (12.5-13.0 %)	1220/3315	1.41	(1.23, 1.61)	1.39	(1.05, 1.84)	1.43	(1.16, 1.76)	1.31	(1.05, 1.63)
Q4 (13.1-31.6 %)	1234/3038	1.72	(1.43, 2.06)	2.45	(1.79, 3.34)	1.58	(1.15, 2.17)	1.52	(1.24, 1.87)
P for trend			*<0.001*		*<0.001*		*<0.001*		*<0.001*
Sarcopenic obesity defined using appendicular skeletal muscle mass[a] plus body mass index[b]									
Q1 (10.5-12.0 %)	321/2676	1.00	(Reference)	1.00	(Reference)	1.00	(Reference)	1.00	(Reference)
Q2 (12.1-12.4 %)	395/2732	1.15	(0.92, 1.42)	1.07	(0.76, 1.56)	0.99	(0.74, 1.32)	1.42	(0.96, 2.10)
Q3 (12.5-13.0 %)	685/3315	1.83	(1.49, 2.23)	1.83	(1.26, 2.66)	1.84	(1.41, 2.39)	1.60	(1.17, 2.20)
Q4 (13.1-31.6 %)	710/3038	2.22	(1.79, 2.77)	2.57	(1.81, 3.65)	2.04	(1.42, 2.93)	2.05	(1.55, 2.73)
P for trend			*<0.001*		*<0.001*		*<0.001*		*<0.001*

[a]Low muscle mass defined as appendicular skeletal muscle mass <29.36% for males and <22.41% for females.
[b]Obesity defined as body mass index ≥30kg/m^2.
Adjusted for age, sex, race/ethnicity, education, household income, smoking pack-years, moderate-to-vigorous physical activity, hypertension, diabetes, cardiovascular disease, C-reactive protein, and hemoglobin (Model D in Table 3).
P for interaction by age = 0.189 for sarcopenia and 0.569 for sarcopenic obesity.
